# Acute Caffeine Intake Does Not Modulate Active or Reactive Inhibition in Non‐Clinical Young Adults, and ADHD Symptoms Do Not Moderate Its Effects

**DOI:** 10.1002/hup.70067

**Published:** 2026-10-04

**Authors:** Ahmet Altınok

**Affiliations:** ^1^ Department of Psychology University of Groningen Groningen the Netherlands; ^2^ Department of Psychology Pamukkale University Denizli Türkiye

**Keywords:** attention‐deficit/hyperactivity disorder, bayesian null evidence, caffeine, cognitive control, crossover trial, preregistration, response inhibition

## Abstract

**Background:**

Caffeine is consumed for its presumed cognitive benefits, yet controlled studies have repeatedly failed to find effects on inhibitory control. Cross‐sectional work suggests that people with elevated attention‐deficit/hyperactivity disorder (ADHD) symptoms may benefit from chronic, moderate caffeine consumption, but whether acute intake improves cognition in proportion to symptom severity has not been tested experimentally.

**Aims:**

We tested whether 250 mg of caffeine improves active inhibition (withholding a prepared response) and reactive inhibition (resolving response conflict), and whether ADHD symptom severity moderates these effects.

**Methods:**

In a preregistered, randomised, double‐blind, placebo‐controlled crossover trial, a nonclinical sample of 36 young adults received 250 mg of caffeine or placebo in counterbalanced order, with testing 45 min after ingestion. Active inhibition was assessed with a Go/No‐Go task (204 trials), reactive inhibition with an arrow flanker task (200 trials) and symptoms with the Adult ADHD Self‐Report Scale. Mixed‐effects models included treatment, symptom severity, their interaction and ten covariates; Bayes factors quantified evidence for the null.

**Results:**

Both tasks produced their benchmark effects (dz = 1.24 and 1.09) and all indices were reliable (split‐half *r* = 0.73–0.98). Caffeine affected neither commission errors (BF01 = 4.98), response criterion (5.14), Go reaction time (2.83), reaction time variability (4.35), flanker reaction time (5.49) nor flanker accuracy (5.09). ADHD symptoms moderated no effect (15 preregistered tests; smallest *p* = 0.514), yet predicted performance itself (flanker accuracy *r* = −0.42), indicating that the null is not due to an insensitive measure.

**Conclusions:**

Caffeine produced no acute benefit for either form of inhibition, and ADHD symptoms did not moderate its effects. Bayes factors supported the absence rather than the nondetection of these effects, arguing against an acute improvement of inhibitory control that scales with ADHD symptom severity in nonclinical young adults. Whether this also holds for chronic consumption or for adults with diagnosed ADHD remains to be tested.

## 1 Introduction

Caffeine is the most widely consumed psychoactive substance in the world, and it is consumed overwhelmingly for its presumed cognitive benefits. Its principal mechanism is antagonism of adenosine A1 and A2A receptors, which prevents adenosine from inhibiting the release of dopamine, noradrenaline and other neurotransmitters in regions supporting attention and executive control (McLellan et al. 2016). On this basis, caffeine has been expected to improve the same functions targeted by stimulant medication. The empirical picture is less uniform than the mechanism suggests. Reviewing the acute literature, McLellan et al. (2016) concluded that doses between roughly 32 and 300 mg reliably enhance alertness, vigilance, reaction time and attention, whereas effects on memory and higher‐order executive function are inconsistent and remain unclear. Effects also depend on the participant's baseline arousal, in line with the inverted‐U relationship between arousal and performance, so that the same dose may benefit a fatigued individual and impair a well‐rested one. This baseline dependency has led to the view that caffeine, particularly when consumed regularly at moderate doses, is better characterised as a normaliser of brain function than as a psychostimulant in the conventional sense: its benefits are most consistently observed when function is compromised, for example, by fatigue, sleep loss or cognitive decline, and are small or absent when performance is already near optimal (McLellan et al. 2016; Nehlig 2010). Effects on memory illustrate this pattern, being more reliably observed when memory function is degraded than in unimpaired individuals. Regular consumption also engages processes that extend beyond the acute receptor blockade. In habitual coffee drinkers, coffee intake reduced resting‐state connectivity within the posterior default mode network, with a similar trend after caffeine alone, a pattern the authors interpreted as increased readiness for action (Picó‐Pérez et al. 2023). In the mouse hippocampus, chronic caffeine intake lowered metabolism‐related processes while inducing neuron‐specific epigenetic changes at genes involved in synaptic transmission and plasticity and enhancing experience‐driven transcription, a profile compatible with an increased readiness to process information (Paiva et al. 2022). These findings distinguish the neural consequences of regular, moderate consumption from those of a single dose, a distinction that is central to the question addressed here.

This inconsistency is most visible for inhibitory control. Tieges et al. (2009) distinguished active inhibition, the deliberate suppression of a prepared or ongoing action, from reactive inhibition, the resolution of interference arising from a competing response, and tested both after a 3 mg/kg dose of caffeine, approximately 210 mg for a 70‐kg adult. Across three double‐blind, placebo‐controlled within‐participants experiments using an AX‐version of the continuous performance test, a stop task and a flanker task, they concluded that caffeine does not modulate inhibitory control. Effects on overall flanker performance and on selective response suppression were negligible; in the stop task, caffeine produced a global change in processing speed rather than any effect specific to inhibition; and although AX‐CPT performance was consistent with improved inhibition, the authors noted that this could equally reflect changes in attentional processes. Barry et al. (2007) reported a comparable dissociation using the same 250‐mg dose employed here, in a randomised, double‐blind, placebo‐controlled crossover study with an auditory Go/No‐Go task. Caffeine reduced reaction time but affected neither omission nor commission errors, and the event‐related potential data showed the same asymmetry: amplitudes of P1, P2 and P3b to Go stimuli increased focally, whereas no component elicited by No‐Go stimuli was affected at all. The authors concluded that caffeine selectively improves processing related to response production rather than acting as a general arousal amplifier. Against this, work using the Attention Network Test has reported dose‐dependent improvements in executive control, reaching asymptote at 200 mg in low habitual consumers but requiring 400 mg in high habitual consumers (Brunyé et al. 2010). Recent work from our own laboratory found no acute effect of 215 mg of caffeine on temporal attention, spatial attention or working memory maintenance, despite all three manipulations producing their expected effects (Akyürek et al. 2025). Taken together, the evidence suggests that caffeine speeds responding without reliably improving the control processes that are usually of theoretical interest, and that habitual intake conditions the dose at which any effect emerges.

A separate literature has proposed that caffeine may be particularly beneficial for individuals with attention‐deficit/hyperactivity disorder (ADHD). The rationale is pharmacological: adenosine A2A receptors are co‐localised with dopamine D2 receptors in dopamine‐rich regions such as the striatum, so blocking them enhances dopaminergic transmission in the same circuits implicated in ADHD, making caffeine a weak analogue of the stimulants used to treat the disorder. More specifically, there is a robust mechanistic basis for expecting caffeine to influence behavioural outputs that depend on the basal ganglia. Striatopallidal A2A receptors, caffeine's principal site of action, interact with dopamine D2, NMDA and metabotropic glutamate receptors to integrate dopaminergic and glutamatergic signalling, exert an overall restraining influence on goal‐directed behaviour and confer homeostatic control over its flexibility (Chen et al. 2023). Because both the withholding of a prepared response and the resolution of response conflict depend on fronto‐striatal circuits, inhibitory control is a natural candidate for such an effect. In a systematic review of 13 animal studies, most using the spontaneously hypertensive rat, Vázquez et al. (2022) concluded that caffeine increases attention and improves learning and memory without altering blood pressure or body weight, although its effects on hyperactivity and impulsivity were contradictory across studies. The authors argued that these cognitive effects might translate to human ADHD, particularly during adolescence.

Human evidence, by contrast, is largely cross‐sectional. Cipollone et al. (2020) drew on the All Army Study of the Army Study to Assess Risk and Resilience in Servicemembers, comparing 1239 soldiers with adult ADHD to 17,674 peers without psychiatric comorbidity. Soldiers with ADHD consumed more caffeine pills, energy drinks and other caffeinated beverages than their peers, and consumption of these products correlated negatively with several individual ADHD symptoms, which the authors interpreted as consistent with a self‐medication account and as pointing to a possible therapeutic role for caffeine in adult ADHD. These associations concern habitual, chronic consumption of moderate amounts of caffeine, not the effect of a single acute dose. The associations were small, however, involved only a subset of symptoms and rested on self‐reported symptom severity rather than performance; the authors themselves concluded that neuropsychological studies are needed to confirm their results. Such designs also cannot distinguish a cognitive benefit of caffeine from selection, expectancy or reverse causation.

The self‐medication account can take two forms. In its affective form, individuals with elevated ADHD symptoms consume caffeine primarily to improve their well‐being, mood or subjective arousal rather than their cognitive performance; this form predicts no particular cognitive benefit and cannot be tested with performance measures. In its cognitive form, which has been used to motivate a possible therapeutic role for caffeine in adult ADHD (Cipollone et al. 2020; Vázquez et al. 2022), caffeine compensates for ADHD‐related difficulties in cognitive control, and this form makes a specific and testable prediction: the acute cognitive effect of caffeine should be larger in individuals with more ADHD symptoms. This is a moderation hypothesis, and it is plausible on more general grounds. Baseline‐dependency is a recurring feature of stimulant effects, and the inverted‐U account of caffeine's action implies that individuals further from optimal arousal should benefit most. ADHD symptom severity in nonclinical samples is continuously distributed and is associated with poorer performance on the very tasks used to index inhibitory control. To our knowledge, however, the hypothesis has not been tested experimentally: no placebo‐controlled study has examined whether ADHD symptom severity moderates the acute cognitive effects of caffeine in nonclinical adults. The present study tests the cognitive form of the account; it does not address the affective form.

The present study addressed this gap in a preregistered, randomised, double‐blind, placebo‐controlled crossover trial. Young adults from a nonclinical sample, varying continuously in ADHD symptom severity, received 250 mg of caffeine or a matched placebo in counterbalanced order and completed a cognitive battery 45 min later. We report here two tasks that met a measurement‐adequacy criterion defined before hypothesis testing, and which between them span the distinction drawn by Tieges et al. (2009): a Go/No‐Go task, in which a prepared response must be deliberately withheld, indexing active inhibition; and an arrow flanker task, in which interference from competing flankers must be resolved, indexing reactive inhibition. ADHD symptoms were measured dimensionally with the Adult ADHD Self‐Report Scale, and both the total score and the inattention and hyperactivity/impulsivity subscales were examined. Following the preregistration, we predicted that caffeine would improve performance and that improvement would be larger at higher symptom levels.

Two features of the analysis deserve emphasis at the outset. First, because both predictions concern the presence of an effect, evidence for their absence requires more than a nonsignificant *p* value; we therefore report Bayes factors quantifying the evidence for the null hypothesis alongside conventional tests. Second, difference scores derived from cognitive tasks are frequently unreliable even when the underlying group‐level effects are robust, the reliability paradox described by Hedge et al. (2018). Karvelis and Diaconescu (2025) have shown that this problem is not confined to correlational analyses of individual differences: observed effects are attenuated in proportion to the square root of measurement reliability whether the comparison is correlational or between groups, so an unreliable index compromises any inference drawn from it. We therefore report split‐half reliability for every index and applied a uniform measurement‐adequacy criterion before hypothesis testing. Three further preregistered tasks did not meet this criterion and are reported descriptively in Supporting Information S1: Appendix A rather than entering the hypothesis tests.

## 2 Method

### 2.1 Participants

Thirty‐six young adults were recruited from university campuses by convenience sampling. The sample was nonclinical in the sense that recruitment neither targeted nor excluded any diagnostic group: participants were drawn from the general student population, and ADHD symptoms were measured dimensionally rather than used as an inclusion criterion. Self‐reported diagnostic history and medication use were recorded; participants reporting a previous diagnosis or regular medication use were retained, and a sensitivity analysis excluding them is reported in Supporting Information S1: Appendix B. Eligibility required the ability to comply with a 24‐hr caffeine abstinence protocol before each session. The preregistered stopping rule specified data collection until 36 participants had completed both sessions; participants completing only one session or providing unusable data were to be replaced. All 36 participants in the final sample completed both sessions. Sample characteristics are reported in Table 1. Participants gave written informed consent and the study was conducted in accordance with the Declaration of Helsinki.

**TABLE 1:** Participant characteristics (*N* = 36).

Characteristic	*M* or *n*	SD or %
Age (years)	22.29	2.68
Women	17	47.2%
Placebo‐first sequence	18	50.0%
Habitual caffeine intake (mg/day)	76.7	62.7
Sleep quality (1–10)	6.47	1.92
ASRS total	51.47	11.73
ASRS inattention	25.78	6.18
ASRS hyperactivity/impulsivity	25.69	6.60

*Note:* Habitual caffeine intake was estimated from an ordinal frequency item. Age was missing for one participant.

Abbreviation: ASRS = Adult ADHD Self‐Report Scale.

### 2.2 Design

The study used a within‐participants, double‐blind, placebo‐controlled, randomised crossover design with two sessions. In one session, participants consumed 250 mg of pure caffeine powder; in the other, they consumed 250 mg of lactose powder as a matched placebo. In both sessions, the powder was dissolved in 200 ml of decaffeinated Nescafé Dolce Gusto capsule coffee (Aromatic Arabica flavour, Lungo serving size, 6/10 intensity) brewed in a Krups KP1208 coffee machine, following the procedure used in previous work from our laboratory (Akyürek et al. 2025), so that volume, temperature, taste, colour and aroma were identical across conditions.

Blinding was maintained by separating three roles across three people. The author generated the allocation sequence in Microsoft Excel, assigning each participant to one of two orders labelled only by arbitrary letters, and took no part in the experimental sessions. The mapping of letters onto caffeine and placebo was known solely to a second researcher, who prepared and served the beverage according to the sequence and had no further contact with the participant. A third researcher received the participant in the laboratory, delivered the task instructions and ran the session; this researcher neither prepared the beverage nor observed it being consumed and remained unaware of the assigned condition throughout. Participants were likewise unaware of which substance they had received. Because the letter code was not disclosed to the author, all data processing and statistical analysis were carried out blind to condition, and the code was broken only after the analysis pipeline had been finalised.

Treatment order was assigned by block randomisation at the participant level and was perfectly counterbalanced (18 participants per sequence) and balanced across sex. Sessions were scheduled at comparable times of day; the mean absolute difference in start time between a participant's two visits was 1.1 hr, and 24 of 36 participants were tested within 1 hr of the same clock time across sessions.

### 2.3 Procedure

Participants were asked to abstain from all caffeine‐containing products for 24 hr before each session. On arrival, they completed a demographic questionnaire and the ASRS and then consumed the assigned beverage. During the 45‐min absorption interval, they waited in a separate waiting area and were asked to occupy themselves quietly; most read or used their mobile telephones. Cognitive testing began 45 min after ingestion and took place on a desktop computer in a quiet laboratory room. The Go/No‐Go task was programmed in OpenSesame (Version 4.1.6) and the flanker task in PsychoPy. Each task was preceded by on‐screen instructions and a practice block.

### 2.4 Measures

#### 2.4.1 Adult ADHD Self‐Report Scale

ADHD symptom severity was measured with the 18‐item ASRS, administered once. Items are rated on a 5‐point frequency scale. Three indices were computed: a total score, an inattention subscale (Items 1–4 and 7–11) and a hyperactivity/impulsivity subscale (Items 5–6 and 12–18). Internal consistency was high for the total score (*α* = 0.90) and both subscales (*α* = 0.85 and 0.83). The standard Part A screening algorithm was applied for descriptive purposes.

#### 2.4.2 Response Inhibition: Go/No‐Go Task

Single uppercase letters were presented sequentially at the centre of the screen. Participants responded to every letter except X, which required withholding the response. The stimulus set comprised ten Go letters (A, D, G, I, J, K, O, R, W and Y) and one No‐Go letter (X). Following 7 practice trials, the experimental block comprised 204 trials, of which approximately one third were No‐Go trials (*M* = 68.0, range 64–72). The response deadline was 900 ms. Preregistered indices were the commission error rate (responses on No‐Go trials divided by the number of No‐Go trials) and sensitivity d′, computed as *z* (hit rate) − *z* (false‐alarm rate) with a log‐linear correction. Omission error rate, response criterion c and mean reaction time on correct Go trials were also analysed, as the preregistration specified both accuracy and reaction time as outcome measures for each task. Three further indices routinely reported for this paradigm were analysed as exploratory outcomes: intra‐individual reaction time variability (the standard deviation of correct Go latencies), its coefficient of variation and post‐error slowing.

#### 2.4.3 Conflict Processing: Flanker Task

An arrow flanker task was administered but was not included in the preregistration; all flanker analyses are therefore labelled exploratory. Participants reported the direction of a central arrow flanked by four arrows pointing in the same (congruent) or opposite (incongruent) direction. Following 8 practice trials, participants completed 200 experimental trials divided into two blocks of 100. The blocks differed in congruency proportion: Block A contained 40 congruent and 60 incongruent trials, and Block B contained 60 congruent and 40 incongruent trials. The congruency effect was computed as the difference in mean reaction time between incongruent and congruent trials. Data from one participant's first session were unavailable owing to a technical failure, leaving 71 participant‐sessions.

#### 2.4.4 Additional Preregistered Tasks

Three further tasks listed in the preregistration, a speeded rapid serial visual presentation task, an n‐back task and a Stroop task, were administered but were found, after data collection, to have been implemented with parameters that departed from validated protocols. They did not meet the measurement‐adequacy criterion defined below and were therefore excluded from all hypothesis tests. Full details, including implementation parameters (Supporting Information S1: Table A1), manipulation checks, reliability estimates and condition‐wise descriptive statistics, are reported in Supporting Information S1: Appendix A.

### 2.5 Data Preparation

Practice trials were removed. Reaction time analyses were restricted to correct trials; latencies below 200 ms were discarded as anticipatory, and remaining latencies more than 3 SD above or below the participant's mean for that task and condition were excluded. This procedure removed 10 flanker trials (0.07%) and no Go/No‐Go trials. No participant met the preregistered exclusion criterion of at‐ or below‐chance overall accuracy.

Split‐half reliability was estimated for each index by computing it separately over odd‐ and even‐numbered trials within each participant‐session, correlating the halves and applying the Spearman–Brown correction. A measurement‐adequacy criterion was applied before hypothesis testing: tasks whose benchmark manipulation check failed, and indices whose corrected split‐half reliability fell below 0.60, were not entered into hypothesis tests. This criterion was applied uniformly across all tasks and outcomes.

### 2.6 Statistical Analysis

Binary trial‐level outcomes were analysed with generalised linear mixed models (logit link for error rates, probit link for signal‐detection models) and reaction times with linear mixed models. Fixed effects were the task condition (trial type in the Go/No‐Go task, congruency in the flanker task), treatment, ADHD symptom severity and their interactions, together with session, treatment order, sex, age, habitual caffeine intake, sleep quality, previous ADHD diagnosis, other psychiatric diagnosis, medication use and caffeine consumption in the preceding 24 hr. Continuous predictors were grand‐mean centred. Random effects comprised by‐participant intercepts and by‐participant slopes for treatment and, where the design permitted, for the task condition and their interaction. Denominator degrees of freedom were estimated with the Satterthwaite approximation.

Participant‐level analyses complemented the mixed models. For each outcome, indices were computed per participant and condition, and caffeine effects were tested with paired‐samples *t* tests; moderation was tested by regressing the caffeine‐minus‐placebo difference score on grand‐mean‐centred ASRS scores with habitual caffeine intake as a covariate, as specified in the preregistration. Bonferroni correction was applied across the five preregistered outcomes.

To quantify evidence for the null hypothesis, Bayes factors (BF01) were computed for every paired comparison and regression coefficient using the Jeffreys‐Zellner‐Siow prior with scale *r* = 0.707. Values above 3 were interpreted as moderate and values above 10 as strong evidence for the null. Robustness of any effect reaching significance was assessed by refitting models with and without covariates and by leave‐one‐participant‐out analyses. The alpha level was 0.05 (two‐tailed).

The study design, hypotheses, sampling plan, outcome indices and analysis plan were preregistered on the Open Science Framework before data collection (https://osf.io/jv974). Deviations from the preregistered protocol are reported below. Data and analysis code are available at the same repository. Analyses were conducted in R (Version 4.3) using lme4 and lmerTest and in Python (Version 3.12).

### 2.7 Deviations From the Preregistration

Five deviations are reported. First, three preregistered tasks (speeded RSVP, n‐back, Stroop) were excluded from hypothesis testing under the measurement‐adequacy criterion described above; they are reported in Supporting Information S1: Appendix A. Second, the flanker task was administered but not preregistered, and all flanker analyses are reported as exploratory and excluded from the correction for multiple comparisons. Third, the preregistration specified exclusion of participants reporting nonadherence to the 24‐hr abstinence protocol; 22 participants reported such consumption on the demographic questionnaire and were retained in the primary analyses because the acute effect was assessed 45 min after administration, with a sensitivity analysis restricted to the adherent subsample reported in Supporting Information S1: Appendix B. Fourth, habitual caffeine intake was recorded with an ordinal frequency item rather than a direct estimate in milligrams per day; ordinal categories were converted to approximate milligram values, and analyses using the untransformed ordinal variable yielded identical conclusions. Fifth, the interval between sessions exceeded the preregistered washout window (median 20 days), which reduced the risk of carryover but allowed greater within‐person variation in state between sessions; treatment order had no effect on any outcome.

## 3 Results

### 3.1 Participants and Symptom Measures

Thirty‐six young adults (19 men, 17 women) completed both experimental sessions. Participants were aged 18–30 years (*M* = 22.29, SD = 2.68); age was missing for one participant and was replaced with the sample mean in the covariate models. Treatment order was perfectly counterbalanced, with 18 participants receiving caffeine in the first session and 18 in the second, and order was balanced across sex (10 men and 8 women in the placebo‐first sequence; 9 men and 9 women in the caffeine‐first sequence). Estimated habitual caffeine intake was low to moderate (*M* = 76.7 mg/day, SD = 62.7), and self‐rated sleep quality on the night before testing was moderate (*M* = 6.47, SD = 1.92). Four participants reported a previous ADHD diagnosis, three reported another psychiatric diagnosis and three reported regular medication use (five participants in total, with overlap between categories); all were retained and these variables were entered as covariates. Because the sample therefore included individuals with a self‐reported diagnosis, we describe it as nonclinical rather than healthy. Excluding these five participants (*n* = 31) did not change any conclusion (Supporting Information S1: Appendix B).

ADHD symptom scores showed adequate dispersion and high internal consistency. Total ASRS scores ranged from 32 to 79 (*M* = 51.47, SD = 11.73, *α* = 0.90). The two subscales were comparable in magnitude (inattention: *M* = 25.78, SD = 6.18, *α* = 0.85; hyperactivity/impulsivity: *M* = 25.69, SD = 6.60, *α* = 0.83) and strongly intercorrelated, *r* = 0.68. Ten participants (27.8%) exceeded the Part A screening threshold for probable adult ADHD. Screening status was not used for selection and is reported only to characterise the distribution of symptoms, which was the dimension of interest. Sample characteristics are reported in Table 1.

### 3.2 Manipulation Checks and Measurement Reliability

Both tasks produced their established benchmark effects. In the Go/No‐Go task, accuracy was substantially lower on No‐Go than Go trials, *M* = 0.138, SD = 0.111, *t* (71) = 10.51, *p* < 0.001, dz = 1.24, and No‐Go accuracy was well below ceiling (*M* = 0.853, range 0.45–1.00), indicating that response inhibition was effectively taxed. In the flanker task, responses were slower on incongruent than congruent trials, *M* = 20.28 ms, SD = 18.64, *t* (70) = 9.17, *p* < 0.001, dz = 1.09; the congruency effect was in the expected direction in 64 of 71 participant‐sessions.

Split‐half reliabilities (odd–even trials, Spearman‐Brown corrected) were adequate to excellent for all indices entered into the analyses: Go reaction time 0.98, omission error rate 0.90, commission error rate 0.84, flanker overall accuracy 0.90 and the flanker congruency effect 0.73.

### 3.3 Main Effect of Caffeine

Caffeine effects were tested with generalised linear mixed models (binary outcomes) and linear mixed models (reaction times), including treatment, ADHD symptom severity and their interaction as predictors of interest, and session, treatment order, sex, age, habitual caffeine intake, sleep quality, previous ADHD diagnosis, other psychiatric diagnosis, medication use and caffeine consumption in the preceding 24 hr as covariates. Random intercepts and random slopes for treatment were included for each participant. Participant‐level descriptive statistics, mean differences, effect sizes and Bayes factors are reported in Table 2; standardised effects are shown in Figure 1 and condition means in Figure 2, 3, 4.

**TABLE 2:** Effects of caffeine on Go/No‐Go and flanker performance.

Outcome	Placebo	Caffeine	*M* diff [95% CI]	*t*	*p*	*p corr*	*dz*	*BF01*
	*M* (SD)	*M* (SD)						
Go/No‐Go (preregistered)								
Commission error rate	0.143 (0.118)	0.150 (0.119)	0.007 [−0.021, 0.035]	0.50	0.622	1.00	0.08	4.98
Omission error rate	0.007 (0.016)	0.011 (0.029)	0.005 [−0.002, 0.011]	1.40	0.169	0.845	0.23	2.27
Sensitivity (d′)	3.65 (0.72)	3.52 (0.72)	−0.128 [−0.320, 0.064]	−1.35	0.185	0.926	−0.23	2.42
Response criterion (c)	−0.65 (0.25)	−0.63 (0.26)	0.016 [−0.061, 0.093]	0.42	0.675	1.00	0.07	5.14
Go RT (ms)	396 (39)	402 (36)	5.8 [−3.9, 15.6]	1.22	0.232	1.00	0.20	2.83
Flanker (exploratory)								
Overall RT (ms)	412.0 (55.7)	411.1 (57.9)	−0.9 [−19.2, 17.5]	−0.10	0.924		−0.02	5.49
Congruency effect, RT (ms)	24.4 (21.7)	16.3 (13.1)	−8.1 [−15.9, −0.3]	−2.10	0.043		−0.36	0.78
Overall accuracy	0.970 (0.031)	0.968 (0.040)	−0.002 [−0.010, 0.007]	−0.42	0.681		−0.07	5.09
Congruency effect, accuracy	0.018 (0.024)	0.022 (0.036)	0.004 [−0.008, 0.016]	0.70	0.490		0.12	4.40

*Note: N* = 36 for Go/No‐Go outcomes and *n* = 35 for flanker outcomes (one participant's first session was lost to technical failure). Reaction time analyses were restricted to correct trials with latencies above 200 ms, after removal of latencies more than 3 SD from the participant's condition mean. The congruency effect is incongruent minus congruent. *p corr* = Bonferroni‐corrected *p* value across the five preregistered outcomes. BF01 = Bayes factor favouring the null hypothesis (JZS prior, *r* = 0.707); values above 3 indicate moderate evidence for the null.

**FIGURE 1:**
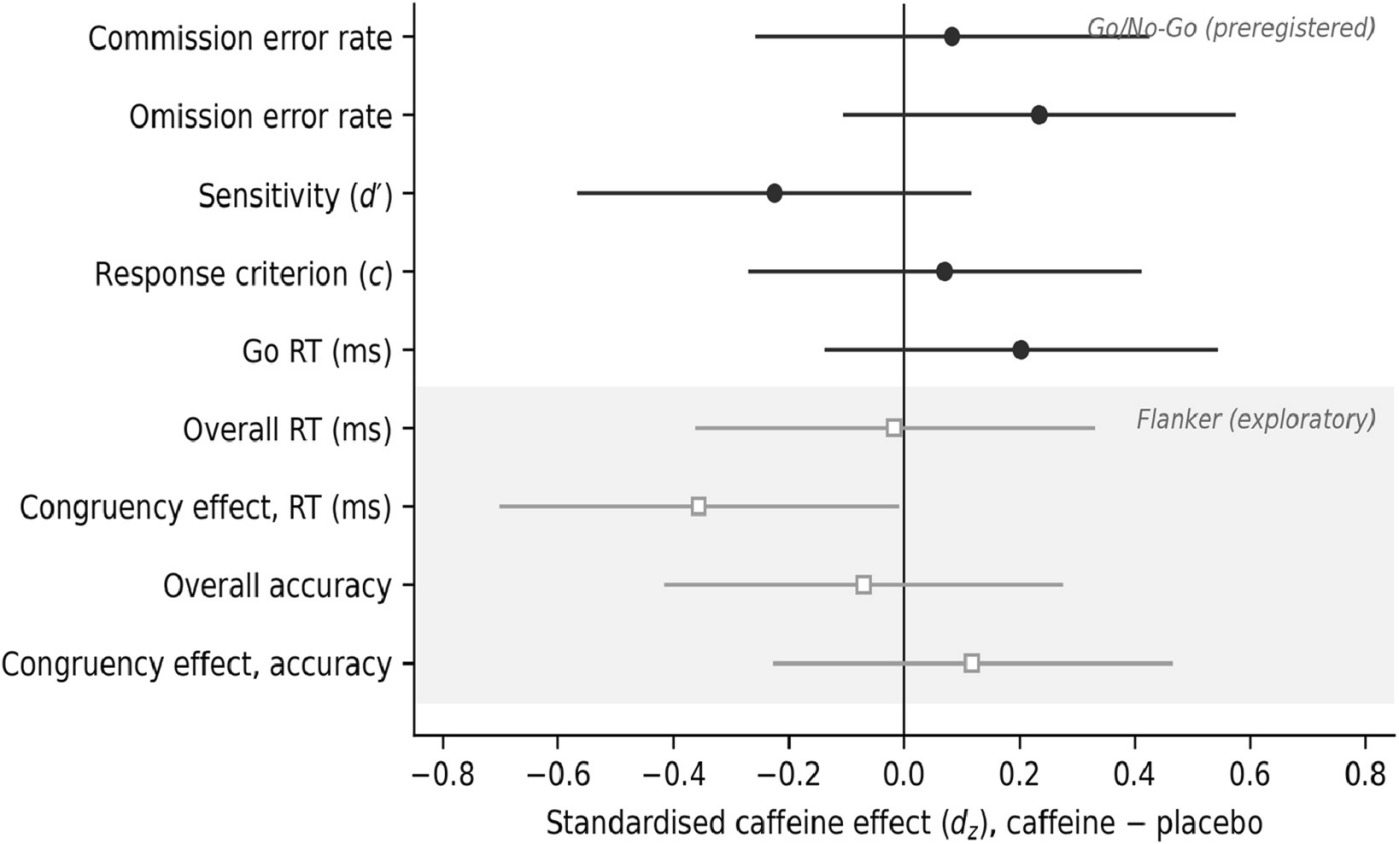
Standardised effects of caffeine on preregistered and exploratory outcomes. Points represent standardised within‐participant mean differences (dz; caffeine minus placebo) with 95% confidence intervals. Filled circles denote preregistered Go/No‐Go outcomes; open squares in the shaded region denote exploratory flanker outcomes. Positive values indicate higher scores following caffeine.

**FIGURE 2:**
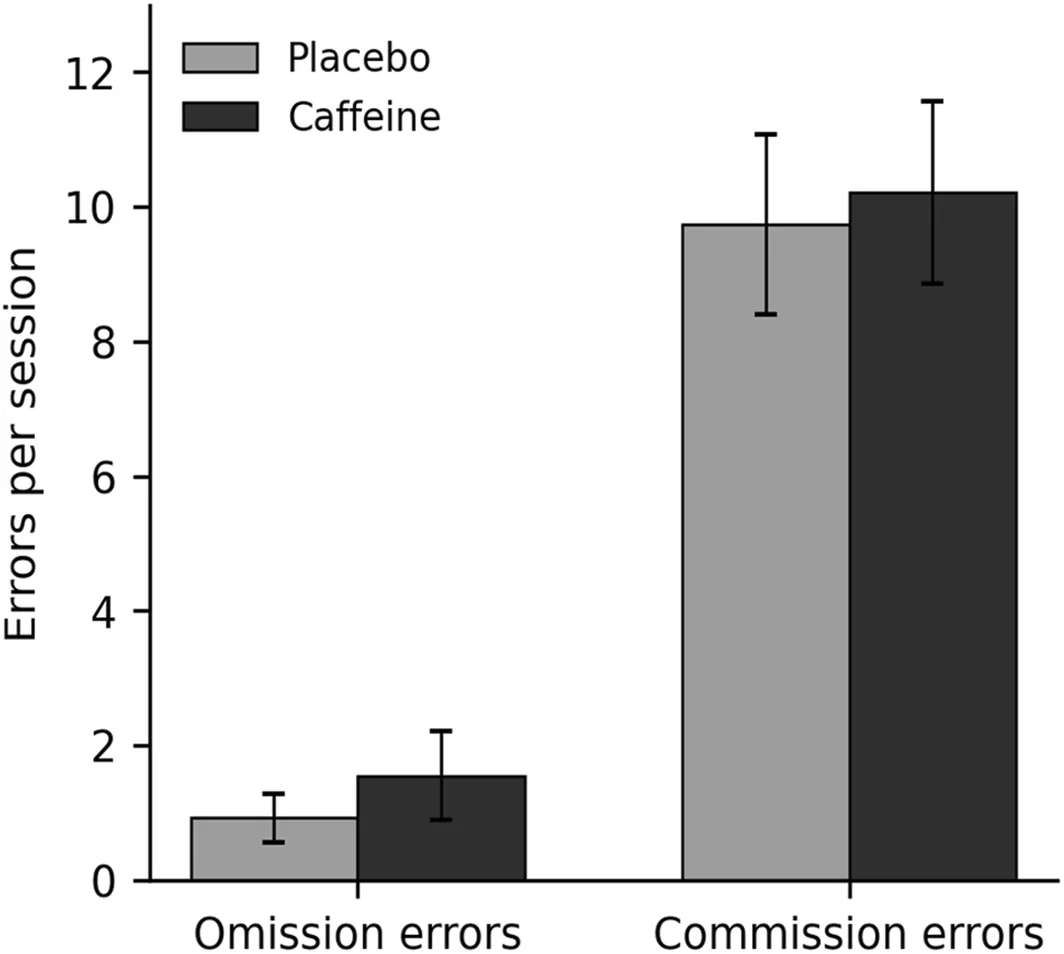
Omission and commission errors in the Go/No‐Go task by treatment condition. Bars show the mean number of errors per session. Omission errors are failures to respond on Go trials (approximately 136 per session) and commission errors are responses on No‐Go trials (approximately 68 per session). Error bars represent standard errors of the mean. *N* = 36.

**FIGURE 3:**
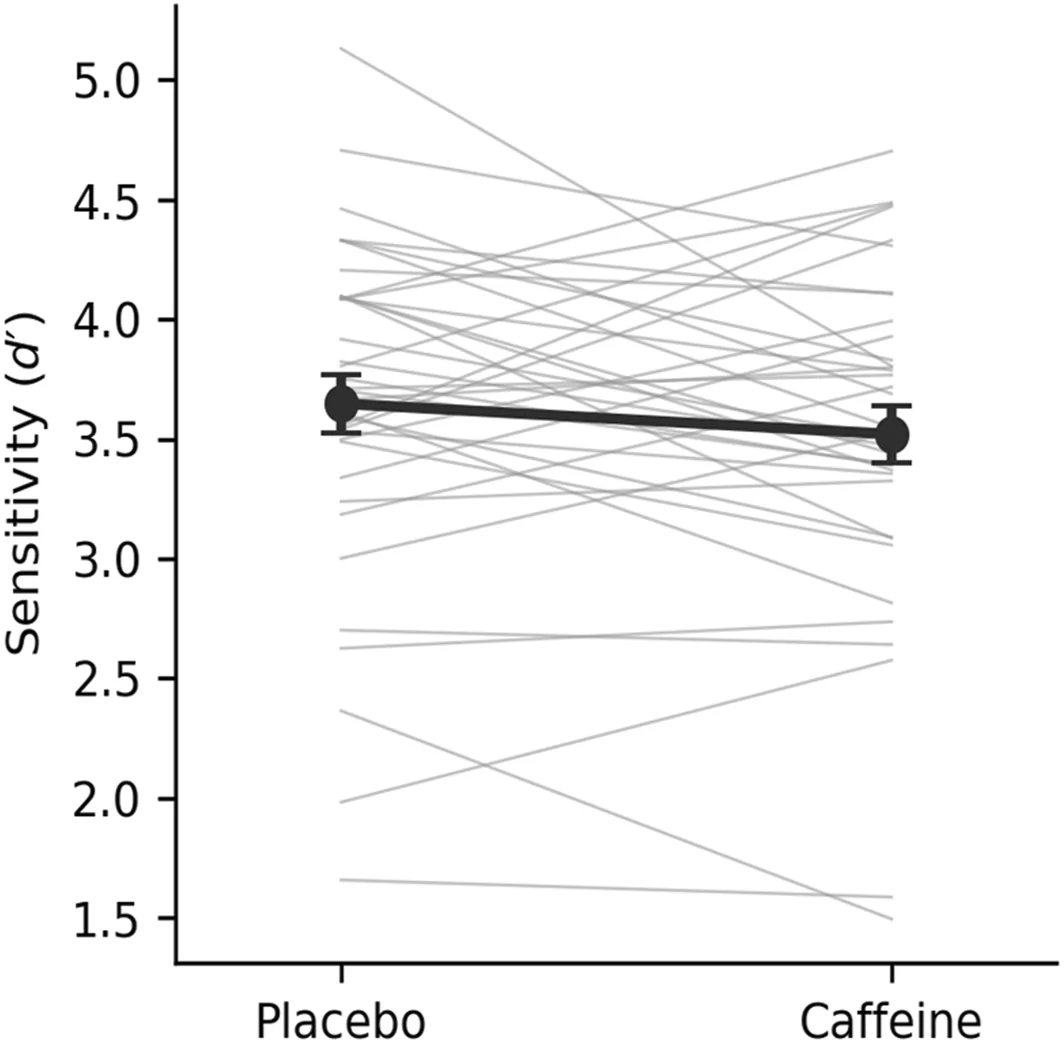
Signal‐detection sensitivity in the Go/No‐Go task by treatment condition. Grey lines show individual participants; the black line shows the group mean with standard errors. *N* = 36.

**FIGURE 4:**
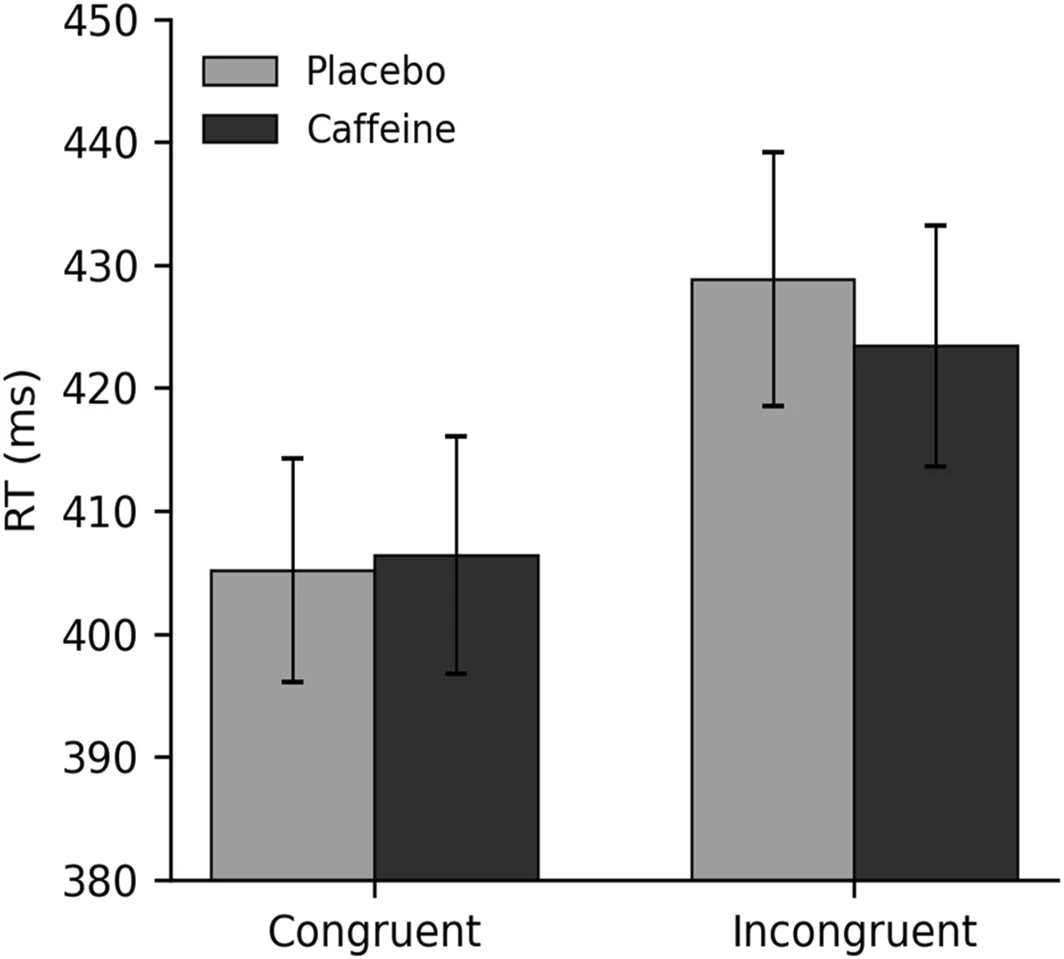
Flanker reaction time by congruency and treatment condition. Bars show mean reaction time for correct responses. Error bars represent standard errors of the mean. *n* = 35.

In the Go/No‐Go task, caffeine did not affect the commission error rate, the primary index of response inhibition. Participants committed a mean of 9.75 commission errors per session under placebo and 10.21 under caffeine (out of approximately 68 No‐Go trials); the mixed model yielded *b* = 0.082, OR = 1.09, 95% CI [0.86, 1.37], *z* = 0.69, *p* = 0.488, and the participant‐level comparison gave Mdiff = 0.007, 95% CI [−0.021, 0.035], *t* (35) = 0.50, *p* = 0.622, dz = 0.08, BF01 = 4.98. Response criterion was likewise unaffected, Mdiff = 0.016, *t* (35) = 0.42, *p* = 0.675, BF01 = 5.14. Go reaction times did not differ between conditions, 396 ms under placebo versus 402 ms under caffeine, *F* (1, 33.0) = 1.29, *p* = 0.265, BF01 = 2.83.

Omission errors were more frequent under caffeine in the fully adjusted model, *b* = 0.587, *z* = 2.02, *p* = 0.043, corresponding to a mean increase from 0.92 to 1.55 errors per 136 Go trials. This effect was not robust. In the model without covariates, it was not significant, *b* = 0.354, *p* = 0.194, and in leave‐one‐participant‐out analyses, it fell below *p* = 0.05 in none of the 36 solutions (range 0.050–0.332). The participant‐level comparison was also nonsignificant, *t* (35) = 1.40, *p* = 0.169, BF01 = 2.27, and the Bonferroni‐corrected *p* value was 0.845.

Signal‐detection analysis indicated lower sensitivity under caffeine. In the probit mixed model, the trial‐type × treatment interaction was significant, *b* = 0.331, *z* = 2.83, *p* = 0.005, and the estimate was stable across model specifications (no covariates *b* = 0.275, *p* = 0.011; adding order *b* = 0.269, *p* = 0.013; adding demographic covariates *b* = 0.266, *p* = 0.014; full model *b* = 0.265, *p* = 0.014). Leave‐one‐participant‐out analyses retained significance in 35 of 36 solutions (*p* range 0.004–0.120). However, the effect was carried entirely by the reduction in Go responding described above (*b* = −0.297, *z* = −2.60, *p* = 0.009) rather than by any change in No‐Go responding, and the participant‐level d′ comparison was nonsignificant, Mdiff = −0.128, 95% CI [−0.320, 0.064], *t* (35) = −1.35, *p* = 0.185, dz = −0.23, BF01 = 2.42.

Two additional Go/No‐Go indices commonly reported in the literature were analysed. Intra‐individual reaction time variability, the standard deviation of correct Go latencies, did not differ between conditions (placebo *M* = 65.26 ms, SD = 20.15; caffeine *M* = 67.01 ms, SD = 20.68), Mdiff = 1.75 ms, 95% CI [−3.09, 6.59], *t* (35) = 0.73, *p* = 0.469, dz = 0.12, BF01 = 4.35, and neither did the coefficient of variation, Mdiff = 0.002, *t* (35) = 0.38, *p* = 0.708, BF01 = 5.23. Both indices were highly reliable (split‐half *r* = 0.92 and 0.91). Post‐error slowing, computed as the difference in Go reaction time following commission errors versus correct rejections, was also unaffected, Mdiff = −4.02 ms, *t* (32) = −0.31, *p* = 0.759, BF01 = 5.14; this index rested on few observations (*M* = 6.2 usable trials per session, range 0–23) and is reported for completeness only.

In the exploratory flanker task, caffeine had no effect on overall reaction time, 412.0 versus 411.1 ms, Mdiff = −0.9 ms, 95% CI [−19.2, 17.5], *t* (34) = −0.10, *p* = 0.924, BF01 = 5.49, or on overall accuracy, Mdiff = −0.002, *t* (34) = −0.42, *p* = 0.681, BF01 = 5.09. The congruency effect was numerically smaller under caffeine (16.3 ms) than placebo (24.4 ms). This difference did not reach significance in the mixed model, *F* (1, 32.7) = 2.91, *p* = 0.097, and the estimate was consistent across analytic approaches (model without covariates *p* = 0.090; participant‐level paired comparison Mdiff = −8.09 ms, 95% CI [−15.90, −0.28], *t* (34) = −2.10, *p* = 0.043, dz = −0.36, BF01 = 0.78). The congruency effect in accuracy was unaffected, Mdiff = 0.004, *t* (34) = 0.70, *p* = 0.490, BF01 = 4.40.

Bayes factors indicated moderate evidence for the null hypothesis for four of the five preregistered Go/No‐Go outcomes (BF01 = 2.42–5.14) and for three of the four flanker outcomes (BF01 = 4.40–5.49). No preregistered outcome remained significant after Bonferroni correction.

### 3.4 Moderation by ADHD Symptoms

ADHD symptom severity did not moderate the effect of caffeine on any outcome. In the mixed models, the treatment × ASRS interaction was nonsignificant throughout: commission errors, *p* = 0.826; omission errors, *p* = 0.114; sensitivity, *p* = 0.116; Go reaction time, *p* = 0.866; flanker reaction time, *p* = 0.638; and the three‐way congruency × treatment × ASRS interaction in the flanker task, *p* = 0.259. The complementary difference‐score regressions, which regressed each within‐participant caffeine‐minus‐placebo difference on grand‐mean‐centred ASRS total scores while controlling for habitual caffeine intake, produced the same pattern (Table 3). Coefficients were close to zero for all five preregistered outcomes (all *p* > 0.57, all Bonferroni‐corrected *p* = 1.00), and Bayes factors indicated moderate evidence against moderation (BF01 = 4.82–5.58). Scatterplots for four representative outcomes are shown in Figure 5.

**TABLE 3:** Moderation of the caffeine effect by ADHD symptom severity.

Outcome and scale	*b*	SE	*t*	*p*	*BF01*
Go/No‐Go (preregistered)					
Commission error rate					
ASRS total	−0.0001	0.0014	−0.05	0.957	5.58
Inattention	0.0010	0.0028	0.34	0.734	5.29
Hyperactivity/impulsivity	−0.0009	0.0024	−0.38	0.704	5.22
Omission error rate					
ASRS total	−0.0001	0.0003	−0.17	0.868	5.51
Inattention	0.0001	0.0007	0.10	0.924	5.56
Hyperactivity/impulsivity	−0.0002	0.0006	−0.36	0.721	5.26
Sensitivity (d′)					
ASRS total	−0.0005	0.0098	−0.05	0.957	5.58
Inattention	−0.0053	0.0191	−0.28	0.782	5.39
Hyperactivity/impulsivity	0.0024	0.0162	0.15	0.884	5.53
Response criterion (c)					
ASRS total	−0.0002	0.0039	−0.05	0.958	5.58
Inattention	−0.0029	0.0077	−0.38	0.704	5.22
Hyperactivity/impulsivity	0.0016	0.0065	0.24	0.814	5.44
Go RT (ms)					
ASRS total	−0.276	0.490	−0.56	0.577	4.82
Inattention	−0.308	0.957	−0.32	0.750	5.32
Hyperactivity/impulsivity	−0.534	0.810	−0.66	0.514	4.56
Go/No‐Go (exploratory)					
Go RT variability (SD)					
ASRS total	0.265	0.246	1.08	0.289	3.27
Inattention	0.490	0.480	1.02	0.315	3.45
Hyperactivity/impulsivity	0.373	0.410	0.91	0.370	3.81
Go RT coefficient of variation					
ASRS total	0.0008	0.0006	1.44	0.160	2.18
Inattention	0.0013	0.0011	1.20	0.240	2.89
Hyperactivity/impulsivity	0.0012	0.0009	1.35	0.188	2.44
Flanker (exploratory)					
Overall RT (ms)					
ASRS total	−0.035	0.937	−0.04	0.971	5.51
Inattention	0.128	1.841	0.07	0.945	5.50
Hyperactivity/impulsivity	−0.182	1.534	−0.12	0.906	5.48
Congruency effect, RT (ms)					
ASRS total	−0.357	0.394	−0.91	0.372	3.77
Inattention	−0.134	0.783	−0.17	0.866	5.44
Hyperactivity/impulsivity	−0.863	0.635	−1.36	0.184	2.37
Overall accuracy					
ASRS total	−0.0005	0.0004	−1.24	0.223	2.72
Inattention	−0.0004	0.0008	−0.46	0.646	4.99
Hyperactivity/impulsivity	−0.0011	0.0007	−1.67	0.105	1.57
Congruency effect, accuracy					
ASRS total	0.0012	0.0006	2.09	0.044	0.80
Inattention	0.0014	0.0012	1.24	0.222	2.71
Hyperactivity/impulsivity	0.0022	0.0009	2.39	0.023	0.46

*Note:* Coefficients are from ordinary least squares regressions of the caffeine‐minus‐placebo difference score on the grand‐mean‐centred ADHD scale, controlling for grand‐mean‐centred habitual caffeine intake (df = 33 for Go/No‐Go, df = 32 for flanker). All Bonferroni‐corrected *p* values across the 15 preregistered tests equalled 1.00 and are therefore omitted. BF01 = Bayes factor favouring the null hypothesis; values above 3 indicate moderate evidence for the null.

**FIGURE 5:**
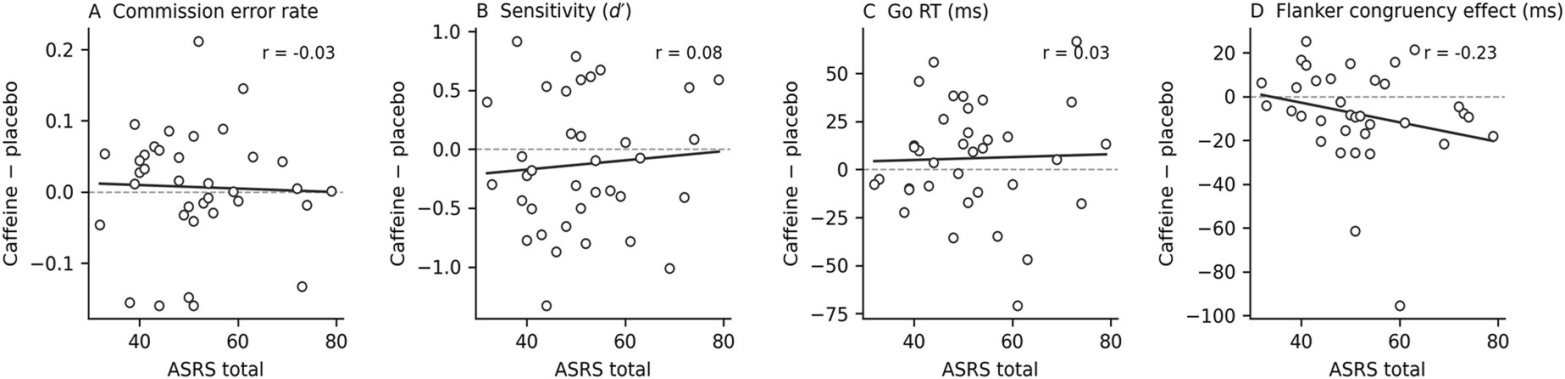
Caffeine effect as a function of ADHD symptom severity. Each point represents one participant's within‐participant difference score (caffeine minus placebo) plotted against ASRS total score, with the ordinary least squares regression line. Dashed lines indicate no difference between conditions.

The same conclusion held when the two subscales were entered separately. Across 18 subscale tests, no coefficient reached the corrected threshold. The smallest uncorrected *p* values were obtained for hyperactivity/impulsivity predicting the caffeine effect on the flanker congruency effect in accuracy, *b* = 0.0022, *p* = 0.023, BF01 = 0.46, and on flanker overall accuracy, *b* = −0.0011, *p* = 0.105, BF01 = 1.57; both concern exploratory outcomes and neither survives correction for the number of tests conducted. Within the preregistered Go/No‐Go outcomes, no subscale coefficient approached significance (all *p* > 0.21).

Habitual caffeine intake was not a significant covariate in any model (all *p* > 0.26) and its inclusion did not alter the ADHD coefficients. Treatment order was likewise nonsignificant throughout (commission errors, *p* = 0.902; omission errors, *p* = 0.832; Go reaction time, *p* = 0.721; flanker reaction time, *p* = 0.315), confirming that counterbalancing was effective.

### 3.5 Exploratory Analyses

#### 3.5.1 ADHD Symptoms and Task Performance

To establish whether the ASRS was sensitive to individual differences in this sample, symptom scores were correlated with performance averaged across the two sessions (Table 4). Correlations were consistently in the theoretically expected direction: higher symptom scores were associated with more commission errors, lower sensitivity, greater reaction time variability, slower flanker responses and lower flanker accuracy. The strongest associations involved hyperactivity/impulsivity and flanker accuracy, *r* = −0.51, *p* = 0.002, and inattention and flanker overall reaction time, *r* = 0.44, *p* = 0.008. Inattention was also associated with Go reaction time variability (SD, *r* = 0.34, *p* = 0.046; coefficient of variation, *r* = 0.38, *p* = 0.021), consistent with the literature identifying intra‐individual variability as a marker of inattentive symptoms. No correlation survived correction for the 33 tests conducted (all pFDR > 0.06), and the two subscales were too highly intercorrelated (*r* = 0.68) to support claims about their differential contributions.

**TABLE 4:** Correlations between ADHD symptom scores and task performance.

Measure	ASRS total	Inattention	Hyperactivity/impulsivity
Go/No‐Go			
Commission error rate	0.24	0.17	0.26
Omission error rate	0.14	0.10	0.16
Sensitivity (d′)	−0.27	−0.19	−0.30
Response criterion (c)	0.02	0.00	0.03
Go RT	0.08	0.15	0.01
Go RT variability (SD)	0.28	0.34*	0.19
Go RT coefficient of variation	0.34*	0.38*	0.24
Flanker			
Overall RT	0.35*	0.44**	0.21
Congruency effect, RT	−0.16	−0.13	−0.16
Overall accuracy	−0.42*	−0.25	−0.51**
Congruency effect, accuracy	−0.12	−0.24	0.01

*Note:* Pearson correlations between ADHD symptom scores and task performance averaged across the two sessions (*n* = 36 for Go/No‐Go, *n* = 35 for flanker). Asterisks denote uncorrected significance: *p < 0.05, **p < 0.01. No correlation remained significant after false discovery rate correction across the 33 tests reported (all pFDR > 0.06). The two subscales correlated *r* = 0.68.

#### 3.5.2 Covariate Effects

Several covariates predicted performance independently of treatment. ADHD symptom severity was associated with a higher omission error rate, *b* = 0.105, *z* = 2.98, *p* = 0.003, and showed a nonsignificant association with slower Go reaction times, *F* (1, 25.0) = 3.40, *p* = 0.077. Sleep quality predicted faster Go reaction times, *F* (1, 25.0) = 4.89, *p* = 0.036, as did caffeine consumption in the preceding 24 hr, *F* (1, 25.0) = 4.39, *p* = 0.046. Sleep quality also predicted lower reaction time variability, *b* = −5.37, *t* (25.0) = −2.42, *p* = 0.023, and a lower coefficient of variation, *b* = −0.009, *t* (25.0) = −2.18, *p* = 0.039. In the flanker task, sex was associated with reaction time, *F* (1, 24.8) = 4.81, *p* = 0.038, and sleep quality showed a marginal association, *p* = 0.062. Reaction time variability was associated with ADHD symptom severity at the participant level (coefficient of variation, *r* = 0.34, *p* = 0.044; standard deviation, *r* = 0.28, *p* = 0.095). The session was a strong predictor in both tasks, with faster responding in the second session (flanker, *F* (1, 32.5) = 16.32, *p* < 0.001).

#### 3.5.3 Sensitivity Analyses

Twenty‐two participants reported consuming caffeine within 24 hr preceding the demographic assessment, whereas the preregistration specified their exclusion. Because the acute effect was assessed 45 min after administration, these participants were retained in the primary analyses. The magnitude of the caffeine effect did not differ between adherent (*n* = 14) and nonadherent (*n* = 22) participants for any outcome (all *p* > 0.25), and the two groups did not differ in placebo‐session performance (all *p* > 0.07). Restricting the analysis to the adherent subsample yielded the same pattern of conclusions. Full sensitivity analyses are reported in Supporting Information S1: Appendix B. A second sensitivity analysis excluded the five participants who reported a previous diagnosis or regular medication use (*n* = 31). No preregistered outcome was affected by caffeine (all *p* ≥ 0.180), no preregistered moderation test was significant (smallest *p* = 0.261) and the reduction in the flanker congruency effect moved further from significance (*p* = 0.115; Supporting Information S1: Appendix B).

The flanker task used unequal congruency proportions across two blocks (Block A: 40% congruent; Block B: 60% congruent). The proportion‐congruency effect was in the expected direction but nonsignificant, 5.0 ms, *t* (70) = −1.64, *p* = 0.105. Blocks did not qualify any caffeine effect (block × treatment, *p* = 0.843; congruency × block × treatment, *p* = 0.565), and analyses collapsing across blocks yielded the same estimates as block‐wise models. Blocks were therefore combined in all reported analyses.

## 4 Discussion

Acute caffeine did not improve inhibitory control in nonclinical young adults, and it did not do so preferentially in those with higher ADHD symptom levels. Every preregistered moderation test was null, with Bayes factors favouring the null hypothesis in each case, and neither the inattention nor the hyperactivity subscale identified a responsive subgroup. The same picture held for the main effects. A single 250‐mg dose left commission errors, omission errors, sensitivity, response criterion, mean Go reaction time, reaction time variability, overall flanker reaction time and flanker accuracy statistically unchanged. One comparison reached conventional significance in a single specification, a reduction of roughly 8 milliseconds in the flanker congruency effect, and is treated below as a hypothesis rather than a result.

A null result is only as informative as the procedure that produced it, and two objections deserve direct answers. The first concerns the manipulation itself. We collected no subjective measure of alertness or perceived drug effect and therefore cannot demonstrate from our own data that participants experienced the dose. Three considerations bear on this. The dose is well characterised: 250 mg is the dose used by Barry et al. (2007) in a comparable crossover design, and it falls within the range that McLellan et al. (2016) identify as reliably affecting alertness and reaction time. The 45‐minute interval brackets the period of substantial plasma absorption, although it need not coincide with peak concentration for every participant. Most usefully, our data contain an indirect index of pharmacological sensitivity: self‐reported caffeine intake in the preceding 24 hours predicted Go reaction time, indicating that the dependent variables were not indifferent to participants' caffeine state. This does not substitute for a subjective manipulation check, and we return to it as a design requirement below, but it does argue against the strongest form of the objection.

The second concerns the tasks. Both worked as intended and both were measured reliably. The Go/No‐Go task produced a large difference between Go and No‐Go trials, the flanker task produced a robust congruency effect and internal consistency estimates for the retained measures were adequate to excellent throughout. The contrast with the three preregistered tasks excluded in Supporting Information S1: Appendix A is instructive. Those tasks were dropped on a single a priori criterion, namely a failed manipulation check or a split‐half reliability below the threshold we had set, and their failures were parametric rather than conceptual. The n‐back task, run with forty trials and no practice block, produced a highly significant load effect alongside a sensitivity index whose reliability was close to zero; the Stroop task, with sixteen critical trials, produced substantial interference with a reliability far below any usable standard. This is the reliability paradox in operation (Karvelis and Diaconescu 2025): group‐level effects that are unambiguous, resting on individual‐level estimates that are essentially noise and therefore incapable of supporting a within‐subject drug comparison. The measures we retained do not have this property.

The central preregistered question returned an unusually clean null across every test, with moderate Bayesian evidence for the null hypothesis in each one. This gains force from a complementary observation: symptom severity was not inert in this dataset. It predicted performance in theoretically coherent directions, with higher symptom scores associated with lower flanker accuracy, slower flanker responses and greater variability in Go reaction time. The last of these is notable, since elevated intra‐individual reaction time variability is among the most consistently replicated performance markers in ADHD (Kofler et al. 2013), and its emergence in a nonclinical sample scoring across the subclinical range suggests that the instrument captured the construct it was intended to capture. Two constraints apply. None of these correlations survived correction for multiple comparisons, so they are underpowered exploratory associations offered as evidence of measurement validity rather than as findings. The two subscales were also strongly correlated with one another, which precludes attributing any association to one symptom domain over the other. What they establish is narrow but sufficient: the moderation nulls did not arise because the moderator was noise.

These results bear on a cross‐sectional literature. Cipollone et al. (2020), analysing Army STARRS data from more than a thousand individuals with ADHD and a much larger comparison group, found caffeine consumption negatively correlated with some ADHD symptoms, an association concerning habitual rather than acute consumption, and called explicitly for neuropsychological studies to establish whether this reflects a cognitive effect. The present experiment addresses that call for the acute effect of caffeine on inhibitory control in a nonclinical sample, and the answer is negative. It does not address chronic consumption or adults with a clinical diagnosis of ADHD.

Two reconciliations remain open. The cross‐sectional association may reflect selection or self‐medication, in the sense that individuals who find caffeine subjectively helpful consume more of it, or it may depend on chronic rather than single‐dose exposure. Neither possibility is addressed by our design. Alternatively, the relevant outcome may not be inhibitory control at all. Subjective symptom relief, sustained attention over longer intervals, or arousal regulation could show effects invisible to a laboratory inhibition battery. This possibility is consistent with the view that individuals with ADHD symptoms consume caffeine to improve their well‐being rather than their cognition, the affective form of the self‐medication account distinguished in the Introduction, which our design was not intended to test and which our results leave untouched. The animal literature reviewed by Vázquez et al. (2022) is consistent with this partition: across the studies they surveyed, attention and learning benefits were reasonably reliable, whereas findings for hyperactivity and impulsivity were contradictory. Our results fall on the contradictory side of that divide.

The scope of the conclusion should be stated precisely. We studied a nonclinical sample varying in subclinical symptom severity, receiving one dose on one occasion. We cannot speak to diagnosed ADHD, to clinical dose ranges or to chronic consumption.

Our inhibition nulls are not anomalous. Tieges et al. (2009) administered 3 mg/kg of caffeine across three experiments using the AX‐CPT, stop‐signal and flanker tasks, a battery designed to separate active from reactive inhibition, and found no reliable caffeine effects on either. Our two tasks span the same distinction, with the Go/No‐Go task indexing action restraint and the flanker task indexing interference control, and we converge on the same conclusion in both. Akyürek et al. (2025) similarly reported null cognitive effects at a comparable dose.

The closest correspondence is with Barry et al. (2007), who used the same 250‐mg dose in a double‐blind crossover with an auditory Go/No‐Go task, and whose behavioural result we replicate on the error side: neither omission nor commission errors were affected. Their electrophysiological data suggest a mechanism. Caffeine enhanced the P1, P2 and P3b components elicited by Go stimuli while leaving every No‐Go component untouched, an asymmetry indicating that the drug amplified processing of stimuli requiring a response while sparing the inhibitory system itself. If caffeine acts on stimulus registration and response mobilisation rather than on the suppression of a prepared response, our commission error null is the behavioural signature of that dissociation and is theoretically expected rather than merely uninformative.

Where we diverge is on response speed. Barry et al. (2007) found faster reaction times alongside unchanged error rates, and Rogers et al. (2013) reported motor speeding in both low and medium‐to‐high consumption groups. We observed neither. The most plausible explanation lies in trial composition. With sixty‐eight of two hundred and four trials requiring withholding, our No‐Go proportion was roughly one third, well above the ten to twenty per cent typical of Go/No‐Go designs. A high No‐Go rate weakens the prepotency of the Go response and encourages proactive caution, and our participants responded accordingly: mean Go reaction time under placebo fell well inside a 900‐ms response window, indicating strategically controlled rather than maximally fast responding. Where response speed is set by a criterion the participant maintains deliberately, a pharmacological effect on motor readiness has little room to express itself. The auditory task used by Barry et al. (2007), with fewer trials and a different Go to No‐Go ratio, imposed materially different demands.

Three features of our sample and protocol converge on the same conclusion: this was not a design in which caffeine's cognitive effects would be expected to be large. The first is habitual consumption. Our sample was weighted towards low consumers, and under the framework advanced by Rogers et al. (2013) on the basis of a large sample, this is precisely the group for whom cognitive benefits are least expected. In their study, caffeine improved mental performance in medium‐to‐high consumers, whereas in low consumers, it did little beyond reducing sleepiness, with anxiety and jitteriness offsetting any gain. The withdrawal‐reversal account (James and Rogers 2005) supplies the mechanism: much of the apparent benefit of acute caffeine reflects the reversal of an overnight abstinence state rather than net enhancement, and a sample with little withdrawal to reverse should show little benefit. We tested habitual consumption by treatment interactions directly and found none, but with a narrow consumption range that test had little power and should not be read as evidence against the account. Two qualifications matter. Rogers et al. (2013) found motor speeding in both groups, so this explanation covers our cognitive nulls but not our absent reaction time effect. And a majority of participants reported caffeine intake in the 24 hours before a session, although sensitivity analyses comparing compliant and noncompliant participants revealed no differences on any outcome.

The second is dose. The obvious alternative, that 250 mg was simply too little, is difficult to sustain. Brunyé et al. (2010) found that consumers with low habitual intake reach a performance plateau at approximately 200 mg, with 400 mg required before high‐intake consumers show comparable gains, so our low‐consumption sample was dosed at or above its expected plateau. The inverted‐U relationship described by McLellan et al. (2016) and Nehlig (2010) raises the opposite possibility, namely that 250 mg overshot the optimum in relatively caffeine‐naïve individuals, producing a level of arousal that cancelled any processing benefit. Having collected no subjective state measures, we cannot adjudicate between an absent effect and an offsetting one. The ecological status of the dose also deserves comment. The relationship between caffeine and health is nonlinear: regular, moderate consumption is associated with the largest benefits, with summary estimates indicating the greatest risk reduction at around three to four cups a day, whereas benefit diminishes and potential harm emerges at higher intakes (Poole et al. 2017). A single 250‐mg dose corresponds to roughly three espresso coffees taken in immediate succession, which is not a typical pattern of consumption among nonpsychiatric adults or adults with ADHD. The present design therefore tested a single, relatively high acute dose rather than the regular, moderate intake to which the putative benefits of caffeine, and the cross‐sectional associations with ADHD symptoms, primarily refer. A null effect at this dose does not speak to the effects of habitual moderate consumption, and the acute dose–response relationship in individuals with elevated ADHD symptoms remains to be characterised.

The third is baseline alertness. Caffeine's best‐replicated effects concern alertness and vigilance, and are largest when alertness is compromised (McLellan et al. 2016). Our participants were young, tested during the day and largely well rested. Our covariate analyses lend this weight while constraining a task insensitivity reading: self‐reported sleep quality predicted Go reaction time, reaction time variability and its coefficient of variation. The measures unmoved by 250 mg of caffeine tracked spontaneous variation in participants' arousal state; they were sensitive to state‐related variance and simply did not register the manipulation. This also identifies the design most likely to detect an effect, namely testing under fatigue or sleep restriction rather than in the rested daytime protocol we used.

Caffeine reduced the flanker congruency effect by approximately 8 milliseconds. This reached conventional significance in a person‐level analysis, but not in the model without covariates and not under the maximal random‐effects structure appropriate to these data (Barr et al. 2013). We regard it as nonsignificant.

We report it because it is stable in direction across specifications and points somewhere theoretically specific: reduced conflict cost with unchanged overall reaction time and accuracy would indicate more efficient reactive interference control rather than a general speed or arousal effect. Three cautions apply. The flanker task was not preregistered, so this analysis carries none of the protection afforded our confirmatory tests. The estimate hovers near the conventional threshold across specifications, which is the signature of an effect that may not exist. And our two blocks implemented a proportion‐congruent manipulation (Gratton et al. 1992) that we collapsed for analysis, so the reported effect averages over conditions recruiting different levels of proactive control, a feature that could dilute a real effect or generate an apparent one. The appropriate next step is a preregistered replication with the block structure preserved as a factor and the congruency effect designated as the primary outcome.

### 4.1 Limitations

We administered a single dose at a single time point and can characterise neither the dose–response function nor the time course. We collected no subjective measure of alertness, anxiety or perceived drug effect; consequently, we cannot verify the manipulation directly, assess blinding integrity or test the arousal‐offset mechanism described above. The washout interval deviated substantially from the preregistered window, with a median of twenty days between sessions, which reduced carryover risk but increased within‐person state variability across sessions, although order effects were null. Caffeine abstinence was violated by a majority of participants. Sleep was assessed with a single item, a coarse instrument for a variable that predicted three of our outcomes. Demographic fields collected within the task environment proved unusable, and all demographic variables were therefore taken from the questionnaire. Finally, the sample was nonclinical and included a small number of participants with a self‐reported diagnosis; although excluding them did not alter any conclusion, the findings do not extend to adults with clinically diagnosed ADHD, in whom the acute effects of caffeine remain to be tested.

Statistical power bounds the claim we can make. With a sample of thirty‐six, the design was sensitive to moderate effects, whereas caffeine effects on cognitive outcomes typically fall well below that threshold. We were therefore not powered to detect the smallest plausible effects, and our Bayes factors are correspondingly moderate rather than strong: several provide reasonable support for the null hypothesis, but the evidence for omission errors and sensitivity is weak. We claim the absence of a moderate effect on inhibitory control in this population, not the absence of any effect.

Finally, the loss of three preregistered tasks yields a methodological recommendation we consider generalisable. Each produced a highly significant group‐level manipulation check while yielding individual‐level estimates with near‐zero reliability. A significant manipulation check is therefore not sufficient evidence that a task can support within‐subject drug comparisons. Piloting should estimate split‐half or test–retest reliability under the exact parameters intended for the main study, and reliability should serve as a preregistered inclusion criterion. Had we applied that standard at piloting rather than at analysis, three tasks would have been reparameterised rather than discarded.

### 4.2 Conclusion

A single 250 mg dose of caffeine did not detectably alter active or reactive inhibitory control in nonclinical young adults, and ADHD symptom severity did not moderate its effects on any preregistered outcome. This held despite a symptom measure that predicted task performance in expected directions and tasks that were both effective and reliably measured. These findings constrain one interpretation of the cross‐sectional association between caffeine consumption and ADHD symptomatology: whatever underlies that association in nonclinical young adults, it does not appear to be an acute improvement in inhibitory control. Whether the same holds for chronic, moderate consumption or for adults with diagnosed ADHD remains to be tested.

## Author Contributions

**Ahmet Altınok:** conceptualisation, methodology, software, formal analysis, investigation, data curation, writing, original draft, writing, review and editing, visualisation, project administration.

## Funding

The author has nothing to report

## Ethics Statement

This study was approved by the Ethics Committee of the Faculty of Humanities and Social Sciences, Pamukkale University, Türkiye (approval number 26345, granted April 2025). Approval was obtained before the start of data collection. All procedures were carried out in accordance with the ethical standards of the institutional research committee and with the 1964 Declaration of Helsinki and its later amendments. Written informed consent was obtained from every participant before enrolment, after the procedures, the substances administered, the associated risks and the right to withdraw at any time without penalty had been explained.

## Conflicts of Interest

The author declares no conflicts of interest.

## Supporting information


Supporting Information S1

## Data Availability

The study protocol, hypotheses, sampling plan, outcome indices and analysis plan were preregistered on the Open Science Framework before data collection and are publicly available at https://osf.io/jv974. The trial‐level and participant‐level data, the analysis scripts used to generate every reported result and the code used to produce all figures are openly available in the same repository. No part of the dataset is restricted; participants consented to the public sharing of anonymised data, and the deposited files contain no identifying information.
