# Supplementary material for: Acute Caffeine Intake Does Not Modulate Active or Reactive Inhibition in Non‐Clinical Young Adults, and ADHD Symptoms Do Not Moderate Its Effects
Source: Hum Psychopharmacol. 2026 Oct 4;41(6):e70067. doi: 10.1002/hup.70067 (PMC13635470; doi:10.1002/hup.70067)
Supplement: Supplementary file 1 [file HUP-41-e70067-s001.docx]

**Appendix A**

**Preregistered Tasks Excluded From Hypothesis Testing**

Three tasks listed in the preregistration — a speeded rapid serial visual presentation (RSVP) task, an n-back task, and a Stroop task — were administered but did not meet the measurement-adequacy criterion defined in the Method and were therefore excluded from all hypothesis tests. This appendix reports their implementation parameters, manipulation checks, reliability estimates, and condition-wise descriptive statistics. No inferential test of caffeine effects or of moderation by ADHD symptoms is reported for these tasks.

The three tasks were administered with parameters that departed substantially from validated implementations, in each case in the direction of a shortened or simplified version (Table A1). Trial numbers were between one third and one fifth of those used in the source paradigms, and practice was minimal or absent.

**Table A1**

*Implementation Parameters of the Excluded Tasks Relative to Typical Published Implementations*

| Task | Present implementation | Typical implementation | Manipulation check |
| --- | --- | --- | --- |
| Speeded RSVP | 75 trials, 2 practice; SOA 215 ms (200 ms item + 15 ms ISI); Lags 2, 3, 8 = 430, 645, 1720 ms | 300 trials, 30 practice; SOA 90 ms; Lags 2, 3, 8 = 180, 270, 720 ms | Failed |
| N-back | 40 trials (20 per load), no practice; 2-back targets 1–7, 3-back targets 8 | 50–156 trials per load, 20–30 practice; fixed target proportion | Passed |
| Stroop | 32 trials (8 word reading, 8 colour naming, 16 colour–word: 4 congruent, 12 incongruent); fixed block order | 72–240 trials; balanced congruency; counterbalanced blocks | Passed |

*Note.* SOA = stimulus onset asynchrony; ISI = interstimulus interval. Lag values in milliseconds denote the interval between the onset of the first and second target. Typical implementations are those used in the source paradigms and in previous work from our laboratory (Altınok et al., 2023; Akyürek et al., 2025).

**Speeded Rapid Serial Visual Presentation Task**

The task required identification of two digit targets embedded in a stream of letter distractors, with the second target reported under time pressure and the first reported at the end of the trial. The demonstration version of the task was administered in error: stimuli were presented for 200 ms with a 15 ms interstimulus interval, yielding a presentation rate of 4.7 items per second, and the experimental block comprised 75 trials preceded by 2 practice trials. Lags 2, 3 and 8 therefore corresponded to target separations of 430, 645 and 1720 ms. The same paradigm implemented at 90 ms SOA in two previous samples from our laboratory produced a reliable attentional blink (Altınok et al., 2023; Akyürek et al., 2025).

The manipulation check failed. Conditional second-target accuracy did not recover at the long lag: T2|T1 accuracy was .869 (SD = .192) at Lag 2, .878 (SD = .191) at Lag 3, and .840 (SD = .203) at Lag 8. The blink magnitude, defined as Lag 8 minus Lag 2, was numerically reversed, M = −.028, SD = .152, t(71) = −1.58, p = .118, and was in the expected direction in only 28 of 72 participant–sessions. Split-half reliability of the blink magnitude was .34.

First-target accuracy also showed an atypical pattern, declining from .962 (SD = .051) at Lag 2 and .951 (SD = .059) at Lag 3 to .704 (SD = .192) at Lag 8. Because the first target was reported at the end of the trial, the long-lag condition required a substantially longer retention interval, and conditioning second-target accuracy on first-target accuracy therefore rested on a smaller and differently selected subset of trials at Lag 8 (M = 18.3 trials, range 7–30) than at Lag 2 (M = 24.0 trials).

A lag effect was nonetheless present in reaction time. Second-target latencies were 756.8 ms (SD = 148.7) at Lag 2, 738.1 ms (SD = 160.2) at Lag 3, and 657.6 ms (SD = 120.5) at Lag 8; the Lag 2 minus Lag 8 difference was M = 94.5 ms, SD = 68.0, t(70) = 11.70, p < .001, dz = 1.39. This indicates that the task remained sensitive to temporal processing constraints in the speed domain, but it does not constitute a canonical attentional blink, and split-half reliability of the reaction-time-based index was .11. Descriptive values by treatment condition were: T2|T1 accuracy .882 (placebo) versus .850 (caffeine); second-target reaction time 706.4 ms versus 742.5 ms.

**N-Back Task**

Participants monitored a stream of letters and responded when the current letter matched the one presented two or three positions earlier. The task comprised 40 trials in total, a 20-trial 2-back block followed by a 20-trial 3-back block, with a 1,000 ms response window and no recorded practice phase. The number of targets was fixed at eight in the 3-back block but varied across participants in the 2-back block (M = 3.0, range 1–7); in 25 of 72 participant–sessions the 2-back block contained two or fewer targets. The letter stream was continuous across the block transition, so that the first three trials of the 3-back block referred to letters presented in the preceding 2-back block.

The manipulation check passed. Accuracy, indexed as hit rate minus false-alarm rate, was .483 (SD = .309) at 2-back and .220 (SD = .198) at 3-back, and the load effect was reliable, M = .262, t(71) = 6.22, p < .001, dz = 0.73. Sensitivity was above chance at both loads (2-back d′ = 1.47, SD = 0.72; 3-back d′ = 0.76, SD = 0.66). Responding was conservative overall (2-back hit rate .570, false-alarm rate .087; 3-back hit rate .325, false-alarm rate .104).

The task was nonetheless excluded because the derived sensitivity index did not meet the reliability criterion: split-half reliability of 3-back d′ was .08, and of the combined index .16. Descriptive values by treatment condition were: 2-back accuracy .441 (placebo) versus .525 (caffeine); 3-back accuracy .262 versus .179.

**Stroop Task**

The task followed the classic three-condition structure and was administered in a fixed block order: 8 word-reading trials (colour names printed in black), 8 colour-naming trials (coloured dots), and 16 colour–word trials in which colour words appeared in congruent (n = 4) or incongruent (n = 12) ink. Responses were made by key press with a 2,000 ms deadline. Blocks were not counterbalanced, so that condition was confounded with practice.

The manipulation check passed. Reaction times were 876.5 ms (SD = 152.7) for word reading, 792.8 ms (SD = 114.7) for colour naming, 858.8 ms (SD = 181.5) for congruent and 944.8 ms (SD = 156.5) for incongruent colour–word trials. The preregistered interference index (incongruent minus congruent) was M = 86.0 ms, SD = 154.0, t(71) = 4.74, p < .001, dz = 0.56; interference relative to the colour-naming baseline was larger, M = 152.0 ms, SD = 143.2, t(71) = 9.00, p < .001. Accuracy followed the expected ordering (word reading .969, colour naming .924, congruent .944, incongruent .868).

The task was excluded because the preregistered interference index rested on only four congruent trials per session and did not meet the reliability criterion (split-half r = .16 for the incongruent-minus-congruent contrast and .56 relative to the colour-naming baseline). Word-reading latencies exceeded colour-naming latencies, the reverse of the classic ordering, which is consistent with a practice effect in the first block. Descriptive values by treatment condition were: interference 91.1 ms (placebo) versus 81.0 ms (caffeine).

**Summary**

One task (speeded RSVP) failed its manipulation check outright; two tasks (n-back, Stroop) produced their benchmark effects at the group level but yielded difference-score indices whose internal consistency was too low to support tests of individual differences or of within-participant treatment effects. All three were excluded before hypothesis testing under a single, uniformly applied criterion. Because the preregistered hypotheses concerning temporal attention, working memory and cognitive inhibition depended on these indices, those hypotheses could not be tested in the present study.

**Appendix B**

**Sensitivity Analyses**

**Caffeine Abstinence**

The preregistration specified exclusion of participants who reported non-adherence to the 24-hr caffeine abstinence protocol. Twenty-two of 36 participants reported consuming caffeine within the preceding 24 hr on the demographic questionnaire. Because the acute effect was assessed 45 min after administration, these participants were retained in the primary analyses and the following checks were conducted.

First, the magnitude of the caffeine effect did not differ between adherent (n = 14) and non-adherent (n = 22) participants for any outcome (all p > .25). Second, the two groups did not differ in placebo-session performance on any outcome (all p > .07), indicating that residual caffeine did not contaminate the placebo baseline. Third, restricting the analysis to the adherent subsample reproduced the pattern of conclusions, with all preregistered Go/No-Go outcomes remaining non-significant. Fourth, self-reported consumption in the preceding 24 hr was entered as a covariate in all models; it predicted faster Go reaction times, F(1, 25.0) = 4.39, p = .046, but did not alter any treatment or moderation estimate.

It should be noted that the demographic questionnaire was administered once rather than at each session, so this item indexes reported consumption at the time of assessment rather than verified adherence on each testing day.

**Habitual Caffeine Intake**

Habitual intake was recorded with an ordinal frequency item and converted to approximate milligram values (M = 76.7 mg/day, SD = 62.7). It was not a significant predictor in any model (all p > .26), did not correlate with the magnitude of the caffeine effect on any outcome (|r| ≤ .21, all p > .21), and analyses using the untransformed ordinal variable yielded identical conclusions.

**Model Specification**

Effects reaching significance in any model were refitted with and without covariates and subjected to leave-one-participant-out analyses. The increase in omission errors under caffeine was significant in the fully adjusted model (p = .043) but not in the model without covariates (p = .194), and it fell below p = .05 in none of the 36 leave-one-out solutions (p range .050–.332). The trial-type × treatment interaction in the signal-detection model was stable across specifications (no covariates b = 0.275, p = .011; full model b = 0.265, p = .014) and retained significance in 35 of 36 leave-one-out solutions, but was carried entirely by the omission effect described above. The flanker congruency × treatment interaction was p = .090 without covariates, p = .097 with covariates, and p = .043 in the participant-level paired comparison.

For within-participant interaction terms, random slopes for the interaction were included where estimable. Omitting these slopes produced substantially smaller p values for the flanker congruency × treatment interaction (p = .010 with by-participant slopes for congruency and treatment only, versus p = .097 with the maximal structure); the conservative maximal-structure estimate is reported throughout.

**Flanker Block Structure**

The flanker task used unequal congruency proportions across two blocks (Block A: 40% congruent, 100 trials; Block B: 60% congruent, 100 trials). Block order was not counterbalanced (40 of 71 participant–sessions began with Block A) and was inconsistent across sessions for 16 of 35 participants. The proportion-congruency effect was in the expected direction but non-significant: the congruency effect was 17.9 ms in the incongruent-weighted block and 22.9 ms in the congruent-weighted block, M = −5.0 ms, t(70) = −1.64, p = .105. Block did not qualify any caffeine effect (block × treatment, p = .843; congruency × block × treatment, p = .565). Split-half reliability of the congruency effect was higher when blocks were combined (.73) than within Block A (.57) or Block B (.39), and analyses are therefore reported with blocks combined.

**Demographic Data Quality**

Sex and age were recorded both on the demographic questionnaire and within the Go/No-Go task. The in-task fields proved unusable: the sex field returned “female” for 34 of 36 participants in the first session and was inconsistent across sessions for 6 participants, and the in-task age field contained implausible values (including 14, 31, 55, and 75 years). All demographic variables reported in this article are therefore taken from the questionnaire. Age was missing for one participant and was replaced with the sample mean in the covariate models; excluding this participant did not alter any conclusion.

**Participants With a Reported Diagnosis or Medication Use**

Four participants reported a previous ADHD diagnosis, three reported another psychiatric diagnosis, and three reported regular medication use; because these categories overlapped, five participants were affected in total. These participants were retained in the primary analyses, with diagnosis and medication use entered as covariates. To establish whether their inclusion influenced the conclusions, all analyses were repeated after excluding them (n = 31; n = 30 for flanker outcomes). The diagnosis and medication covariates were removed from these models because they no longer varied; all other covariates were retained.

The pattern of conclusions was unchanged. At the participant level, caffeine did not affect any preregistered Go/No-Go outcome: commission error rate, Mdiff = .007, t(30) = 0.46, p = .649, BF01 = 4.73; omission error rate, Mdiff = .005, t(30) = 1.29, p = .206, BF01 = 2.45; sensitivity, Mdiff = −.138, t(30) = −1.37, p = .180, BF01 = 2.23; response criterion, Mdiff = .023, t(30) = 0.55, p = .585, BF01 = 4.53; and Go reaction time, Mdiff = 3.2 ms, t(30) = 0.60, p = .551, BF01 = 4.41. Reaction time variability was likewise unaffected (SD, p = .899, BF01 = 5.18; coefficient of variation, p = .965, BF01 = 5.22). In the mixed models, the commission error rate (p = .523) and Go reaction time (p = .591) were unaffected, and the omission error and signal-detection effects observed in the fully adjusted models for the full sample were of the same direction and magnitude (omission errors, b = 0.684, p = .035; trial type × treatment, b = 0.377, p = .004); as in the full sample, neither was supported at the participant level.

In the flanker task, caffeine did not affect overall reaction time (p = .917, BF01 = 5.12) or accuracy (p = .472, BF01 = 4.03). The reduction in the congruency effect under caffeine was of similar magnitude to that in the full sample (−7.2 ms versus −8.1 ms) but further from significance (participant level, t(29) = −1.63, p = .115, BF01 = 1.59; mixed model, p = .156), consistent with the treatment of this effect as non-significant in the main text.

ADHD symptom severity did not moderate the effect of caffeine on any preregistered outcome. Across the 15 preregistered difference-score regressions, the smallest p value was .261, and Bayes factors ranged from 2.87 to 5.22, exceeding 3 in 14 of 15 tests; in the mixed models, all treatment × ASRS interactions were non-significant (p = .062–.924). Among exploratory outcomes, the association between symptom severity and the caffeine effect on the congruency effect in accuracy, which was present at the uncorrected level in the full sample (ASRS total, p = .044), was somewhat stronger (ASRS total, b = .0017, p = .012; hyperactivity/impulsivity, b = .0025, p = .014). Its direction indicates a larger accuracy cost of conflict under caffeine at higher symptom levels, the opposite of the benefit predicted by the self-medication account, and it did not survive correction for multiple comparisons.
